# Leucémie agressive à cellules NK (Natural Killer): à propos d'un cas dans la population africaine et revue de la littérature

**DOI:** 10.11604/pamj.2018.31.28.16360

**Published:** 2018-09-13

**Authors:** Amine Benmoussa, Khaoula Khalil, Fatimazzahra Boufarissi, Ilias Tazi

**Affiliations:** 1 Service d'Hématologie CHU Mohammed VI, Marrakech, Maroc

**Keywords:** Leucémie agressive à cellules NK, syndromes lymphoproliferatifs NK, CD56, pronostic, traitement, Aggressive natural killer cell leukemia, lymphoproliferative syndromes of NK cells, CD56, prognosis, treatment

## Abstract

La leucémie agressive à cellules NK (ANKL) fait partie des syndromes lymphoprolifératifs à cellules NK. C'est une entité rare touchant essentiellement les asiatiques, elle est très rarement décrite dans la population africaine (l'intérêt de notre cas). Nous rapportons le cas d'une patiente de 19 ans, d'origine marocaine, qui présente un syndrome d'insuffisance médullaire d'installation brutale avec syndrome tumoral, le diagnostic de la Leucémie à NK a été retenu par l'étude microscopique et immunophénotyique de la moelle osseuse. L'évolution est fatale avec décès de la patiente dans 2 mois après le diagnostic par un choc septique. Le pronostic d'ANKL est très mauvais et la survie médiane après le diagnostic est en moyenne de deux mois. En l'absence d'étude prospective, aucune attitude thérapeutique consensuelle n'est à ce jour établie.

## Abstract (en)

Aggressive natural killer cell leukemia (ANKL) is a disease entity within the spectrum of lymphoproliferative syndromes of NK cells. It is rare, preferentially affecting Asiatic people. It has been very rarely reported in the African population; hence the interest of our case. Our study involved a 19-year old female patient of Moroccan origin presenting with acute-onset bone marrow failure syndrome associated with tumor syndrome. The diagnosis of natural killer cell leukemia was retained based on microscopic and immunophenotypic study of the bone marrow. Patient's outcome was fatal; she died within 2 months of diagnosis due to septic shock. Patients with ANKL have a worse prognosis and the median survival time following diagnosis is, on average, two months. In the absence of a prospective study, no therapeutic guidelines have been developed.

## Introduction

Les syndromes lymphoprolifératifs à cellules NK représentent des entités très rares (moins de 5% de tous les néoplasies lymphoïdes). Dans la classification de l'organisation mondiale de santé (OMS) des hémopathies lymphoïdes matures, 3 types de tumeurs lymphoïdes à cellules NK ont été proposés en fonction de leurs caractéristiques cliniques et pathologiques distinctes: leucémie agressive à cellules NK, Lymphome NK/T extra-ganglionnaire de type nasal, syndrome lymphoprolifératif chronique à cellules NK. La leucémie à cellules *natural killer* (NK) est due à une expansion clonale de lymphocytes granulaires agressifs non-T CD3-, elle se caractérise par une forte incidence chez les Asiatiques, une forte association avec le virus d'Epstein-Barr (EBV) et une évolution clinique agressive prédominée par la fièvre, altération de l'état général, l'hépatosplénomégalie, la coagulation intravasculaire disséminée et l'hémophagocytose, son pronostic est péjoratif du fait de la résistance des cellules tumorales NK à la chimiothérapie. Il n'y a pas de traitement standard actuellement disponible et la survie rapportée est entre deux et six mois [1-5].

## Patient et observation

Il s'agit d'une patiente âgée de 19 ans, qui présente un syndrome (sd) anémique fait d'asthénie et de pâleur cutanéomuqueuse depuis 21 jours. Le tableau s'aggrave par l'apparition d'un sd infectieux fait de fièvre isolée, et un sd hémorragique fait des taches ecchymotiques diffuses avec gingivorragies de faible abondance, le tout évolue dans un contexte d'altération de l'état général. L'examen clinique trouve une patiente fébrile à 39.5C, consciente avec un score de Glasgow à 15/15^éme^, une tension artérielle à 10/7cmHg, une saturation en Oxygène à 100%, une fréquence cardiaque à 78 bat/min, les conjonctives décolorées, des taches ecchymotiques diffuses, des bulles hémorragiques au niveau de la muqueuse buccale, hépatomégalie avec une flèche hépatique à 15cm, une splénomégalie à 3cm de débord costal, pas d'adénopathies périphériques, le reste de l'examen somatique est normal.

Le bilan biologique montre a l'hémogramme une anémie normocytaire normochrome arégénérative avec Hb (hémoglobine) à 4.2g/dl, VGM (volume globulaire moyen) à 83 femtolitre, TCMH (Teneur corpusculaire en hémoglobine) à 28 et réticulocytes: 25 10^9^/L, une thrombopénie avec un nombre de plaquettes à 6 10^9^/L, une leucopénie avec des globules blancs à 1,9 10^9^/L, des polynucléaires neutrophiles: 0,21 10^9^/L, et des lymphocytes à 1,4 10^9^/L. Le myélogramme est riche avec de rares mégacaryocytes (R+++ M+); Infiltré par 90% des cellules blastiques granuleuses myéloperoxydase négative. L'immunophénotypage médullaire montre des cellules tumorales exprimant le CD7+, CD22+,CD45+, CD56+,HLA-DR+, et n'exprimant pas le CD3-, CD4-, CD5-, CD10-, CD19-, CD20-: aspect est en faveur de leucémie à cellules NK. Le reste de bilan objective une cytolyse hépatique avec une alanine amino-transférase (ALAT) à 98UI/L et une aspartate amino-transférase (ASAT) à 125UI/L, Lacticodéshydrogénase (LDH) est élevée à 390UI/L, Urée: 0,25, créat: 10, fibrinogène: 1,1g/l, sérologies VIH, VHC, EBV, VHB sont négatives. Le bilan radiologique montre une hépatomégalie à 16cm avec splénomégalie à 18cm à l'échographie abdominale, la radio de thorax est normale ainsi que l'échographie cardiaque. La patiente est initialement mise sous transfusions en unités plaquettaires, plasma frais congelé et culots globulaires, une antibiothérapie à base de céphalosporine 3éme génération est démarrée, puis la patiente est mise sous protocole GRAALL 2013, l'évolution est défavorable, la patiente est décédée après deux mois de diagnostic par un choc septique.

## Discussion

La leucémie agressive à cellules NK se définit (selon OMS) par une prolifération des cellules NK au niveau du sang et de la moelle osseuse d'évolution clinique agressive précédée ou pas d'une phase chronique ou d'une atteinte localisée NK. C'est une maladie non encore bien clarifiée du fait de sa rareté et de la complexité de l'ontogenèse des cellules NK [6]. C'est une maladie décrite pour la première fois en 1986,qui touche surtout les sujets d'Asie, d'Amérique centrale et d'Amérique de sud, pourtant dans notre cas présent, la patiente est d'origine africaine, en effet cette répartition géographique particulière est vraisemblablement due à des facteurs génétiques et environnementaux, l'incidence de la maladie est bimodale avec un premier pic chez les adolescents et les jeunes adultes et le deuxième chez les sujets plus de 60 ans. Les causes de la maladie sont inconnues, plusieurs hypothèses sont évoquées principalement virale du fait de l'association fréquente de virus Epstein Barr (EBV) avec ANKL (>90% des cas). Néanmoins, ce n'est pas le cas pour notre patiente qui est EBV négative [7]. Les signes cliniques de la maladie sont dominés par la fièvre qui est presque quasi constante, l'hépato-splénomégalie qui est retrouvée dans près de 83% des cas, la splénomégalie ou l'hépatomégalie isolée ne sont retrouvées que dans 16%. L'adénopathie est présente dans environ 50% des cas ainsi que Le syndrome d'insuffisance médullaire. L'atteinte cutanée prenant des aspects polymorphes n'est présente que dans 30%. D'autres organes et tissus peuvent être infiltrés tels que le système nerveux central SNC par l'expression de CD56, (molécule d'adhésion cellulaire neuronale) par les cellules NK et le rein (soit par l'infiltration tumorale elle-même ou par le traitement). Notre patiente a un syndrome d'insuffisance médullaire et hépato splénomégalie [8]. Sur le plan morphologique, les cellules NK peuvent avoir plusieurs aspects allant d'une cellule ressemblant à un gros lymphocyte granuleux à une cellule atypique avec un noyau de contours irréguliers contenant de grosses granulations cytoplasmiques azurophiles.

Le phénotype des cellules NK anormales peuvent être similaire à celui des cellules NK normales, avec une expression membranaire de CD3e, CD2, CD7, CD11c, CD16 et CD56, et l'absence d'expression de CD3 et CD57. L'ANKL peut parfois avoir un phénotype anormal, permettant de le différencier des cellules NK normales, telles que la perte de CD16, CD56 ou CD7 [9]. À titre comparatif avec notre patiente, présente à l'immunophénotypage médullaire des Cellules tumorales NK exprimant le CD7+, CD22+, CD45+, CD56+, HLA-DR+, et n'exprimant pas le CD3-. Plusieurs anomalies cytogénétiques ont été rapportées dans L'ANKL, telles que les anomalies des chromosomes 6q et 11q [10], qui sont fréquemment rapportées dans la littérature. Le caryotype n'a pas été demandé dans notre cas. Le diagnostic différentiel se pose essentiellement avec d'autres syndromes lymphoprolifératifs NK: le lymphome NK extranodal est caractérisé par une atteinte périphérique touchant des sujets plus jeunes, avec une meilleure sensibilité à la chimiothérapie et la radiothérapie par rapport à L'ANKL. La leucémie à grands lymphocytes granuleux et le syndrome lymphoprolifératif chronique NK sont deux pathologies d'évolution indolente caractérisées par une lymphocytose chronique. Le pronostic d'ANKL est très mauvais avec une médiane de survie après le diagnostic d'environ 58 jours, du fait de l'agressivité de la maladie et la résistance à la chimiothérapie, attribuée à l'expression des cellules NK de P-glycoprotéine. Aucun protocole de chimiothérapie n'est standardisé jusqu'à ce jour. L'amélioration de pronostic des leucémies à cellules NK peut être obtenue par Allo-SCT (allogreffe des cellules souches hématopoïétiques) avec le maintien d'une rémission complète à long terme. Il est extrêmement difficile de programmer une allo-SCT pour ANKL, en raison de la progression rapide de la maladie et la détérioration rapide des malades. Par conséquent, une programmation précoce de l'allo-SCT est indiquée, dès que le diagnostic d'ANKL est posé. Certains régimes contenant de la L-asparaginase et de la gemcitabine ont montré une certaine efficacité thérapeutique, permettant d'obtenir des remissions temporaires en attente d'une allo-SCT [11].

## Conclusion

L'ANKL est une pathologie rare d'évolution fatale. Sa prise en charge n'est pas codifiée d'où l'intérêt d'autres études afin de standardiser le traitement et améliorer son pronostic.

## Conflits d’intérêts

Les auteurs ne declarent aucun conflit d'intérêts.
